# Evolution of X‐ray machine quality control acceptance indices

**DOI:** 10.1120/jacmp.v10i4.3007

**Published:** 2009-10-07

**Authors:** Maria Lucia Nana I Ebisawa, Maria de Fatima A Magon, Yvone M Mascarenhas

**Affiliations:** ^1^ Universidade de Mogi das Cruzes Mogi das Cruzes, São Paulo Brazil; ^2^ Sapra Landauer São Carlos São Paulo Brazil

**Keywords:** X‐ray equipment, quality assurance, dose linearity, applied kilovoltage

## Abstract

The quality assurance (QA) of radiographic images based on the operating conditions of X‐ray equipment is essential for good image quality, accurate medical diagnostics and for the prevention of health professionals and patients to unnecessary doses of ionizing radiation. This paper presents a historical analysis of 1,635 Quality Assurance Technical Reports of Health Institutions in the state of São Paulo, Brazil, over a seven‐year period. Based on acceptable limits for variations between nominal and measured parameters as the variable to determine the quality of X‐ray equipment operating conditions, a significant improvement was found in the percentage of acceptance of the overall parameters described in the QA technical reports over this period. As expected, we found a strong concentration in the categories of conventional and portable X‐ray equipment, which represent 72% and 84%, respectively, of the total number of equipments. A mechanical parameter such as half‐value layer (HVL), which is important not only for image quality but also for radiation protection, showed significant improvements. Again, only 58% of portable X‐ray equipment showed HVL values recommended for 80 kVp (above 2.3 mm Al), a percentage that improved to 76% in 2006. With regard to mammographers, which are newer machines, all the analyzed systems presented acceptable HVL values. Conventional X‐ray machines showed an increase of this conformity index from 89% in 2000 to 94% in 2006. All this improvement was attributed to the continuous and extensive enforcement of Regulation Act 453 in the state of São Paulo. The improvement in equipment quality control standards is expected to result in the improvement of diagnostic quality, as well as in the reduction of exam repetitions, and thereby reducing the patient's exposure to radiation.

PACS number: 87.57.C

## I. INTRODUCTION

The goal of achieving high quality standards today extends beyond regulatory requirements, since it is an intrinsic factor for a company's good standing in the market. The first international standard of quality assurance established in the market was the International Standards Organization 9000 (ISO 9000) which was created in 1987. This first standard was originally designed for manufactured products, but today it encompasses a variety of production processes and services in a wide range of sectors. In the industrial sector, the ISO is considered a differential, ensuring that certified companies follow a standard that guarantees the quality of their operating processes.

To reduce differences in the provision of imaging services around the world, the World Health Organization (WHO) since the 1970s has established criteria for equipment quality to provide basic radiography.^(1)^ Since the 1950s the Pan American Health Organization (PAHO) has addressed the problem of effectiveness and safety in radiology departments in the Americas by performing national and regional surveys and by implementing quality assurance and quality control programs.^(2)^

In Brazil, the Sanitary Surveillance Agency (ANVISA) of the Ministry of Health established a mandatory regulation, Regulation Act 453 (1998), which requires all radiodiagnostic and dental services to implement a quality assurance program (QAP).^(3)^ This QAP includes a periodic quality control of all X‐ray equipment and image processing systems.^(4)^

The establishment of a quality control program implies the implementation of a set of procedures for the regular and periodic testing of medical equipment and the evaluation of imaging quality to ensure the radiodiagnostic imaging process is in conformity with regulations.^(^ ^5^ ^,^ ^6^ ^)^ Health Services and Radiology Sectors use a variety of quality systems and models, including Continuous Quality Improvement (CQI).^(7)^ Due to the strong competitiveness of the sector, business administrators must focus on continuous improvements to reduce costs and enhance the efficiency and quality of these services.

An objective evaluation of radiological image quality is a complex function that involves numerous parameters. This process can be simplified by dividing image quality analysis into two subgroups: the image‐producing system and the image‐recording system. The image‐recording subsystem consists mainly of the conventional imaging system including the intensifying screen radiographic film, film cassette, and processing system. This aspect of quality assurance (QA) was analyzed previously by us in a study of the behavior of sensitometric curves obtained at the Diagnostic Centers in the state of São Paulo, where Sapra Assessoria provided quality control (QC) services and analysis during the period of 2000 to 2004. The results of that study were published in the Annals of the CLAEB – Latin American Congress on Biomedical Engineering 2007,^(8)^ showing the tendency towards an improved quality of processing systems as a consequence of the Regulation Act 453.

For the image formation parameters subset, we emphasized the importance of accuracy and reproducibility of the kVp (kilovolt peak), current and exposure time, beam collimation system, and correct positioning of the antiscatter grid. Constancy tests for the routine quality assurance verification of X‐ray equipment, required as the minimum established by Regulation Act 453, are a good indicator for the evaluation of the image quality of a service and are, therefore, also important for the quality of the diagnosis given by the radiologist who analyzes the images.

In this paper, we present the data and an analysis of the results contained in the technical reports of X‐ray equipment evaluated at Health Services in the state of São Paulo, Brazil, over a seven‐year period by Sapra Assessoria. Only the results of even years are presented here, in order to show clearly the improvements achieved in the quality parameters of X‐ray equipment in response to the establishment of the Regulation Act 453.

## II. MATERIALS AND METHODS

In this study to analyze the evolution of the quality parameters of X‐ray equipment over time in alternate years from 2000 to 2006, we analyzed the reports based on the quality control measurements performed by Sapra Assessoria at health institutions providing radiodiagnostic services in the state of São Paulo, Brazil.

All the equipment utilized by Sapra Assessoria to measure quality parameters are calibrated and certified annually by IPEN/CNEN (Energy and Nuclear Research Institute, National Nuclear Energy Commission) and by IEE‐USP (Electro Technical and Energy Institute, University of São Paulo).

The following equipment and accessories were used for the measurements analyzed in here:

- Radcal Ionization Chamber ‐ Model 3036: to evaluate the linearity and reproducibility of the exposure rate and exposure time exactitude and reproducibility (This equipment is also used to evaluate patient entrance doses in different radiological exams, but is not presented in this paper.)
- RMI aluminum attenuator set and Radcal Ionization Chamber ‐ Model 3036: to evaluate the half‐value layer (HVL) of each X‐ray machine, mainly at 80 kV
- RMI Model 240A: to evaluate the kVp value and exposure time exactitude and reproducibility
- RMI model 161A Beam Alignment test tool and RMI model 162A Collimator Test Tool: to evaluate beam alignment and congruence of light fields and X‐ray fields
- RMI model 112B Radiographic Focal Spot Size: to evaluate focal point assessment

Sapra Assessoria reports are based on field measurements of X‐ray equipment carried out by trained technicians. These technicians take all the established measurements based on a well‐defined protocol prepared by the medical physicists responsible for Sapra Assessoria. All the data are collected and afterwards recorded in a Sapra Assessoria database for posterior analysis and subsequent preparation of QC reports to be submitted to Sapra's clients.

The QC reports analysis of the measurement data are sent by mail to the health service, which has contracted the services of Sapra Assessoria, addressed to person responsible for the equipment. This report includes a final summary of conforming and nonconforming items and recommendations. The nonconforming items must be adjusted and the QC measurements must be undertaken by Sapra Assessoria again for a new QC report.

Up to 1998, the tolerance limits for each evaluated parameter were not clearly defined, although it was recommended that international limits should be adopted for purposes of conformity analyses. After Regulation Act 453 was established, the tolerance limits became well‐established and the current analysis has become more objective.

The measured parameters and analysis were based on the following guidelines:

- Peak kilovoltage of the beam (kVp) – this parameter is directly responsible for the maximum energy of the photon beam produced
- Exposure rate linearity and reproducibility – ensures that the dose rate applied is linear with the current (mGy/mAs) and is constant for a given voltage and focal point distance
- Exactitude and reproducibility of exposure time – ensures the reproducibility of an exam and allows for a choice of mAs combinations, independently of the specific time selected and the current to produce the desired mAs
- Half‐value layer (HVL) – which is defined as the thickness of the absorption material required to reduce the exposure value to half of its original value and determines the mean beam energy value (beam quality)
- Focal spot – evaluates the minimum resolution of the equipment
- Grid alignment – ensures that the center of the grid coincides with the center of the beam and exam table system
- Beam alignment – ensures that the center of the beam is perpendicular to exam table system (film and table)
- Light and X‐ray field congruence – ensures that the expected X‐ray beam field and size is congruent with the light size and field applied

All the equipment under study were analyzed based on the above parameters, since all of them affect the image quality.

## III. RESULTS

Table 1 lists the total number of equipment analyzed grouped into four categories. As expected, we found a strong concentration in the categories of conventional and portable X‐ray equipment, which represent 72% and 84%, respectively, of the total number of equipments.

**Table 1:** Distribution of X‐ray equipments: by category and year of analysis.

*Category*	*2000*	*2002*	*2004*	*2006*
Conventional & Portable X‐ray Equipment	243	274	318	400
Mammography Equipment	36	46	63	69
Radioscopic Equipment	20	29	39	57
Others	1	5	20	25
Total	300	354	440	551

### A. Electronic parameters: kVp, mA and exposure time (see Figs. 1, 2 & 3)

Among the electronic parameters, a significant improvement was found in the conformity of kVp exactitude, mainly in portable systems. At the early time of Regulation Act 453 establishment, only 33% of the portable X‐ray machines analyzed by Sapra Assessoria showed a maximum of 10% variation, which is the upper variation limit allowed, in comparisons of nominal and measured kVp values. This level of conformity increased to 55% in 2002, 74% in 2004, and 81% in 2006. Considering that many of these portable machines are used in pediatric and neonatal centers, these results appear to indicate a drastic reduction in repetitions of exams on patients for whom the risk of ionizing radiation is greater due to their young age.

**Figure 1:**
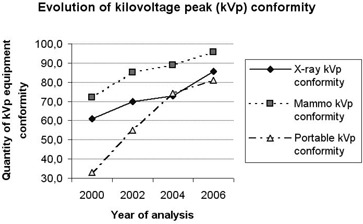
Evolution of kilovoltage peak (kVp) conformity.

**Figure 2:**
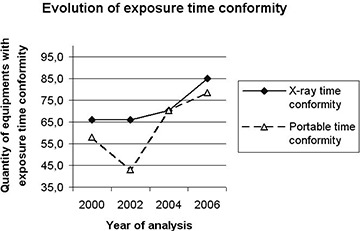
Evolution of exposure time conformity.

**Figure 3:**
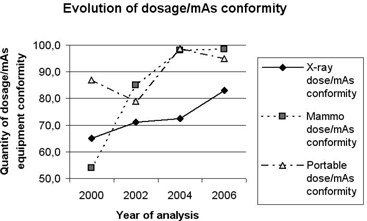
Evolution of dosage/mAs conformity.

Conventional X‐ray equipment also showed an improvement in conformity in comparison with nominal and measured kVp values – 61% of kVp conformity in 2000, increasing to 70% in 2002, 73% in 2004, and 85.5% in 2006. In conventional X‐ray systems, Sapra Assessoria tested at least three current stations per kVp value, and nonconforming results were registered whenever any of the current stations showed a deviation of more than 10% between nominal and measured kVp values, which also applies to any of the three measured kVp values. If we observe the results for only lower current station among others available, disregarding the results for higher current stations (300 and 500 mA), a more significant improvement would be found. However, this was not done because in many cases the higher current stations are actually used in some routine exams, although not as frequently as the 50, 100 and 200 mA stations. For this reason, all stations must be considered in our analysis.

With regard to mAs constancy, which reflects the linear dependence of dose values as a function of the selected time and current station, we highlight our findings on exposure time. Again, in portable X‐ray equipment, which often has only one current station per chosen kVp value, exposure adjustments are done according to the selected irradiation time a significant improvement was detected in the precision of exposure times in this equipment category. The conformity rate was 52% in 2002, improving to 78.5% in 2006. For the conventional X‐ray equipment, conformity in the accuracy of exposure time was 60% in 2000, increasing sharply to 85% in 2006.

### B. Mechanical parameters: HVL, beam alignment, light field congruence, and grid alignment (see Figs. 4 & 5)

A parameter such as HVL, which is important not only for image quality but also for radiation protection, showed significant improvements. Again, 52% of portable X‐ray equipment showed HVL values above the recommended for 80 kVp (2.3 mm Al), a percentage that improved to 76% in 2006. With regard to mammography units, which are newer machines, all the analyzed systems presented acceptable HVL values. Conventional X‐ray machines showed a decrease of this index (nonconformity) from 11% in 2000 to 6% in 2006.

**Figure 4:**
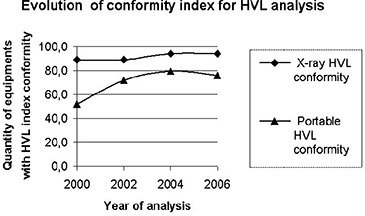
Evolution of conformity index for HVL analysis.

**Figure 5:**
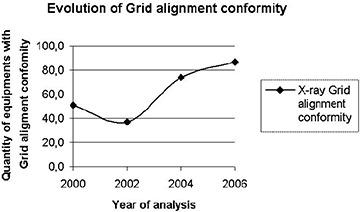
Evolution of the grid alignment conformity index for conventional X‐ray equipment.

The appropriate positioning of the antiscatter grid at exam table system is another very important mechanical parameter. Grid misalignment is responsible for a considerable number of replications of large‐field exams (mainly chest). The grid is used in conventional X‐ray machines and most portable systems do not use the grid during exams. However, this is not the case in orthopedic clinics, where portable machines are mainly used as fixed equipment in a given X‐ray room. In a mammography unit, the grid is adjusted by the manufacturer and no significant misalignments were found. The data analyzed for conventional X‐rays indicated that only 51% of the grids were adequate in 2000; this index decreased to 37%, while 2004 and 2006 presented significant improvements, with the conformity rate increasing to 74% and 86.6%, respectively. The abrupt increase of this index in 2004 reflects a change in the methodology to evaluate grid alignment, as introduced by Sapra Assessoria.

## IV. DISCUSSION

Our analysis based on the Sapra Assessoria reports clearly revealed that the more sophisticated machines, such as fluoroscopes and mammography, showed a very low nonconformity index, indicating that most of the parameters analyzed conform to the current standards of QC measurements and QAPs. This is the purpose of the establishment of regulations such as Regulation Act 453. The greater concern is with the equipment commonly found in diagnostic centers and hospitals (e.g. portable and conventional X‐ray equipment). This category not only represented 76% of all the machines which were analyzed by Sapra Assessoria, but also indicated that older equipment are still used in the majority of radiological examinations. The exams carried out with this equipment are a routine procedure typically used by Brazil's public health system (Sistema Único de Saúde (SUS)). Portable X‐ray equipment is also a cause for concern, since these machines are used extensively in most of hospital Intensive Care units, surgery rooms, and neonatal centers, where the environment frequently lacks protective barriers for workers, other patients in the same room, and the general public. In numerous cases in Brazil, the workers that perform these exams in surgical centers, intensive care units, and patient rooms, do not even have a personal radiation dosimeter for future analysis of radiation exposure and correspondent exposure records. In view of all these important findings and, considering that the state of São Paulo where the economic situation in the capital and the cities of the interior of the state is more homogeneous, our analysis concentrates on the institutions located in the state of São Paulo – a pioneer in the enforcement of Regulation Act 453. The state of São Paulo was the first state to establish and reinforce radiation protection regulations through the state's health department Resolution SS625 of 1994, a state technical rule for protection against ionizing radiation. This resolution was followed by Regulation Act 453, which has a national scope.

The state of São Paulo is the country's economic center located in southeastern Brazil and comprising 22% of Brazil's population (40 million residents in the state of São Paulo in 2004). Statistics show that São Paulo has 10 939 health institutions, almost half of which (5,054) are public health services. The importance of public health can be measured by its budget, which was R$4.9 billion for 2008, corresponding to approximately 10% of the budget of the Ministry of Health, whose total budget for 2008 was R$51.8 billion.^(9)^ This amount represents 15% of the Federal Government's budget for primary expenses, which in turn corresponds to 47.37% of the total budget for federal government expenses in 2008. These facts, allied to the lack of information compiled about the frequency and type of radiological examinations performed in the state, justifies the goal of the present work. The number of X‐ray machines reported by Freitas and Yoshimura^(10)^ was based on 2004 data, which does not differentiate between portable and conventional X‐ray equipment. In this work we have analyzed 9.5% of the total conventional plus portable X‐ray equipment in the state (a total of 3.375 X‐ray machines) and 10.2% of the mammography units (a total of 615 in the state). This represents a significant percentage of the total number of X‐ray equipment in the state of São Paulo.

Our compilation shows unmistakably that the introduction and subsequent enforcement of the regulations imposing radiodiagnostic imaging quality analysis parameters has, from 2000 to 2006, led to significant improvements in the conformity of X‐ray equipment quality control parameters in the state of São Paulo.

The improvement detected can be attributed to a greater awareness by health institutions for the need to adopt a culture of radiation protection, and to demands by society for quality processes, as well as the continuing practice of regulation enforcement on the part of the Sanitary Surveillance Agency (ANVISA).

## V. CONCLUSIONS

There is no question that the observance of the regulations established by Regulation Act 453 and its recommendations has been fundamental to ensure the improvement of quality standards and good working conditions of the X‐ray equipment and image processing conditions at the radiodiagnostic services in the state of São Paulo. Based on the evolution of these parameters, it is also clear that maintenance services have improved, which is reflected by the good adjustment of X‐ray equipment based on the quality control report and recommendations according to Regulation Act 453, which is delivered to the diagnostic center. The improvement in equipment QC standards is expected to result in the improvement of diagnostic quality, as well as in the reduction of exam repetitions, therefore reducing the patient's exposure to radiation. As QA procedures become part of the working culture, compliance with the regulations becomes a natural outcome of the routine at diagnostic centers.

## ACKNOWLEDGEMENTS

The authors gratefully acknowledge the financial support of CAPES and Faep/UMC (Brazil).

## Supporting information

Supplementary Material Files
