# Supplementary figures and images for: Evolution of X‐ray machine quality control acceptance indices
Source: J Appl Clin Med Phys. 2009 Oct 7;10(4):252–9. doi: 10.1120/jacmp.v10i4.3007 (PMC5720561; doi:10.1120/jacmp.v10i4.3007)

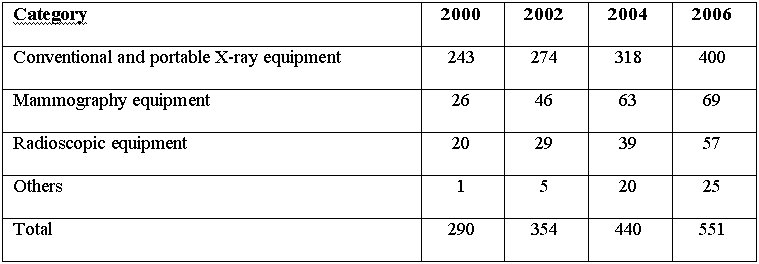

Supplement: Supplementary file 1 — Supplementary Material Files [file ACM2-10-252-s001.jpg]
